# Analytical Study of Temperature Fields in Aluminum Alloy Castings During Solidification in Sand and Metal Molds

**DOI:** 10.3390/ma19091849

**Published:** 2026-04-30

**Authors:** Rostyslav Liutyi, Dmytro Ivanchenko, Andrii Velychkovych, Andriy Andrusyak, Mykhailo Yamshinskij, Ivan Petryk

**Affiliations:** 1 Department of Foundry Productions, National Technical University of Ukraine “Igor Sikorsky Kyiv Polytechnic Institute”, 37, Prospect Beresteiskyi, 03056 Kyiv, Ukraine; rvl2005@ukr.net (R.L.); cortdm77@gmail.com (D.I.); yamshinskiy@ukr.net (M.Y.); 2 Department of Construction and Civil Engineering, Ivano-Frankivsk National Technical University of Oil and Gas, 15 Karpatska Str., 76019 Ivano-Frankivsk, Ukraine; andriiandrusiak@gmail.com; 3 Department of Engineering Mechanics, Engineering and Computer Graphics, Ivano-Frankivsk National Technical University of Oil and Gas, 15 Karpatska Str., 76019 Ivano-Frankivsk, Ukraine; iyap@ukr.net

**Keywords:** aluminum alloy, thermal field, analytical calculation, thermophysical properties, solidification dynamics, casting, foundry

## Abstract

The article presents the calculation of temperature fields for a casting (a cylinder 20 mm in diameter) made of Al–5%wt.Cu alloy, poured into sand (sand–clay) and metal (steel) molds at a temperature of 1123 K (with a metal mold temperature of 523 K). Many existing analytical approaches do not explicitly account for key features such as the time-dependent temperature evolution at the casting surface and center, as well as the variable temperature gradient within the casting. In this paper, the parameters calculated for the sand mold include the surface temperature change over time, as do the dynamics of the solidification front progression, and ultimately, the overall thermal field of the casting. For the metal mold, the process first determines the change in the center temperature over time, followed by the surface temperature dynamics, and finally, the complete thermal field of the casting. Particular attention is paid to determining the position of the mushy zone, namely the zero fluidity and feeding temperatures (the point at which the liquid phase loses mobility upon cooling). These temperatures are critical for casting structure formation and the initiation of shrinkage defects. To perform the calculations, the authors developed original mathematical models and provided solutions to the resulting differential equations. The study demonstrates the differences between the thermal fields in sand and metal molds: the maximum temperature difference is 195 K in the sand mold, compared to 90 K in the metal mold. Therefore, the solidification conditions for this casting in the metal mold are more favorable. The metal mold provides more favorable thermal conditions and a lower analytically predicted tendency toward shrinkage defects, but it does not guarantee their complete absence.

## 1. Introduction

Aluminum alloys are key lightweight structural materials in modern engineering, as they combine low density with high strength, stiffness, and heat-treatment strengthening capability. Consequently, they are highly promising for aerospace, transport, automotive, and other high-tech industries. In foundry production, these alloys offer extensive shaping capabilities for manufacturing critical components with complex geometries [1,2,3,4]. The Al–5%wt.Cu composition is the representative hypoeutectic casting alloy of the Al–Cu system, suitable for analyzing heat transfer, feeding conditions, microstructure evolution, and the formation of the casting’s performance properties during solidification. Its study holds practical significance for increasing the technological reliability of lightweight cast part manufacturing, primarily for aerospace and specialized mechanical engineering [3,4,5].

The formation of metal product properties is determined by their intracrystalline structure. The melt solidification process is the fundamental factor in shaping the microstructure of the metal matrix. Grain structure parameters, dendritic morphology, and the tendency toward porosity or other structural imperfections are driven by the thermophysical interaction within the ‘metal mold’ system during the solidification stage [6,7]. This solidification process is inherently dynamic and non-equilibrium. The ratio of solid to liquid phases changes over time, simultaneously altering the thermophysical properties that dictate the solidification rate [8].

Traditional sand casting of aluminum alloys is gradually giving way to advanced methods, such as squeeze casting, continuous casting, gravity, or pressure die casting; these are some of the casting processes where product quality is affected by casting–mold heat-transfer conditions [9,10,11].

Cast parts, regardless of the manufacturing method—whether in expendable (non-metallic) molds or reusable (metal) molds—undergo three stages of formation: cooling of the liquid metal to the onset of solidification, the solidification process itself, and the cooling of the already solidified metal [8]. Establishing the patterns of temporal temperature distribution during alloy solidification is the key aspect of ensuring the structural integrity and soundness of castings. Consequently, the in-depth study of the thermal field evolution during the solidification of cast parts remains the priority in both scientific and practical research.

In this context, the chemical composition of the metal in liquid parts is never homogeneous, rather than pure metals, alloys of two or more chemical elements are used. Alloys do not have the clearly defined melting point, and their entire solidification process occurs during cooling from the liquidus temperature (*T_L_*) to the solidus temperature (*T_S_*) [12,13,14].

For castings of the Al–Cu system alloys, the initial non-steady-state cooling stage after pouring is decisive, as it dictates the rate of superheat dissipation, the scale of temperature gradients, and feeding conditions—and consequently, the metal’s susceptibility to segregation, porosity, and the formation of the heterogeneous structure [15,16,17,18]. At the same time, the predictive value of thermal calculations at this stage is significantly limited by the uncertainty of heat transfer at the ‘casting–mold’ interface. Here, due to shrinkage, gas gap formation, contact pressure redistribution, and surface state changes, the interfacial heat transfer becomes non-steady and spatially non-homogeneous [19,20,21,22]. For sand molds, this necessitates the correct identification of effective boundary conditions based on temperature measurements [20,21], whereas for metal molds, the interfacial resistance is further influenced by the tool’s own thermal state and the organization of heat dissipation [19,23,24].

In related surface engineering problems, the systematic analysis of the “structure–properties–field” relationship is essential for aluminum–aluminum oxide bilayer systems [25], functionally graded ceramic coatings under localized loading [26], and multilayer or graded architectures intended for service in extreme environments [27]. From a practical standpoint, a more accurate description of thermal fields during solidification supports the controlled reduction in defects and the stabilization of material properties in cast components subjected to intense vibrational and contact loading. This is particularly important for assemblies in which service life and vibration reliability are critical, such as drill bits, shell-type damper components, drilling pump parts, and shock absorbers [28,29,30,31]. Given the demands of aerospace engineering for lightweight structural and heat-resistant materials, improving the accuracy of predicting thermal fields, microstructure, and the properties of cast products remains practically relevant for modern aerospace applications [32,33].

The foundation for analytical modeling of thermal field distribution was laid by J. Stefan in 1890 during his study of soil freezing. According to the law he formulated, the thickness of the solid phase increases proportionally to the square root of the process time. Schwarz adapted these approaches to casting processes using the Gauss error function to describe temperature distribution across the casting and mold sections; however, the significant limitation was the static nature of the contact surface temperature [14,34]. Further development of the heat transfer theory is associated with the work of A. Veinik, who proposed approximate equations for the casting’s thermal field, although their application is limited by the use of empirical constants. The major contribution to the field was made by G. Balandin, who successfully generalized existing knowledge into analytical equations for calculating solidification kinetics in non-metallic molds. These specific solutions are integrated into most modern computer-aided engineering (CAE) systems for foundry production [14,34].

## 2. Theory

Many researchers agree on the importance of the comprehensive assessment of thermal fields in the ‘casting–mold’ system. This is also relevant in light of the development and widespread use of new eco-friendly molding materials, whose thermophysical properties differ from those previously known [35,36,37]. The foundation for the analysis of the entire system is the detailed and accurate determination of the thermal field dynamics of the primary heat source—the casting itself.

For metal molds, accounting for imperfect thermal contact is of fundamental importance: an analytical solution to the problem involving the gas gap and interfacial thermal resistance shows that it is the evolution of thermal resistance at the ‘casting–mold’ interface that can dictate solidification kinetics [38]. Interfacial heat transfer remains the key uncertainty. Therefore, the literature provides the detailed analysis of the physical mechanisms of heat transfer at the contact interface and their influence on heat dissipation intensity [39,40], as well as the development of inverse approaches for reconstructing the heat transfer coefficient at the ‘casting–mold’ boundary based on experimentally measured temperature curves [41,42]. As an example of an analytical formulation specifically oriented toward permanent mold casting, one can cite the two-layer cylinder model that encompasses both the casting and the mold, accounts for the interfacial heat flux, and provides the closed-form solution constructed using Green’s functions; its validity is confirmed by numerical and experimental data [43]. For the practical identification of the heat transfer coefficient in die casting and gravity casting, instrumented molds are used, alongside statistical generalizations of how materials and technological parameters influence interfacial heat transfer intensity [44,45]. The impact of coatings on casting–mold heat exchange and its consequences on the thermal state and microstructure formation have been studied separately [46]. Experimental and replication studies of the high-pressure die casting process clarify the actual thermal boundary conditions within the tooling [47], while axial solidification experiments with aluminum alloys provide direct estimates of the interfacial heat transfer coefficient and thermal diffusivity as input parameters for models [48]. The distinct research area is the optimization of the mold’s thermal regime, particularly through the rational design of cooling channels, which is regarded as the means of controlling cooling non-uniformity [49].

Comparison with sand molds is valid only when identified heat transfer parameters at the ‘casting–sand’ interface are available, as effective boundary conditions here change over time and differ significantly from metal molds in terms of both contact physics and the system’s heat storage capacity [50]. Additionally, it has been shown that the structural design of the sand mold—for example, the use of skeleton patterns—substantially affects the thermal regime, along with the resulting stresses and deformations. Thus, without explicitly accounting for mold parameters, any comparison between sand and metal molds remains methodologically incomplete [51].

With the implementation of the finite element method, material design has transitioned to the level of precision computer analysis. Discretizing the object’s geometry into elementary grid cells has enabled the description of complex process dynamics through the system of approximate equations, providing materials scientists with a powerful framework for predicting product properties during the development stage [52,53,54,55]. The simulation results can be used for optimization of casting process parameters, improving product quality and eliminating casting defects [56]. Despite the widespread use of commercial software packages, the calculated data obtained through them often prove to be inadequate due to the neglect of specific boundary conditions or inaccuracies in the input thermophysical parameters. Most critical input values for such simulation software are the thermo-physical properties of the cast metal and mold, as well as interface boundary conditions. But these values are temperature dependent. Therefore, the values are difficult to acquire for different metal–mold–process combinations. As a result, the outcome of simulation software may deviate from reality [57].

The available calculation methods and the programs built on their basis require precise specification of boundary conditions, one of which is the temperature of the casting surface. In addition, it is necessary to establish the law of its change in time, which none of the existing methods allow us to do. Therefore, for this class of casting problems, the predictive accuracy of numerical temperature-field simulations depends critically on the correct specification or identification of boundary conditions, interfacial heat-transfer parameters, and temperature-dependent thermophysical properties. If these inputs are oversimplified or insufficiently identified, substantial deviations between simulated and actual thermal histories may occur.

The disadvantages of existing calculation methods are that they do not take into account the dynamics of the temperature change in the casting surface. In addition, the calculations often use interdependent values, which significantly reduces the reliability of the results.

In our previous publication [58] devoted to the study of solidification conditions of steel castings, important points were theoretically and practically established. Among them: the time of heat removal from overheating, i.e., the time from the moment of filling the mold to the start of the solidification process (cooling the casting surface to the liquidus temperature T_L_); the time of completion of solidification of the casting surface (cooling it to the solidus temperature); and the dynamics of the solidification front from the surface to the thermal center of the casting, in which the solidification process itself is completed. The uniqueness of this system of mathematical calculations lies in the fact that we took into account the practically real distribution of the temperature field inside the casting during solidification, which was given minimal attention in the scientific and technical literature. The temperature field of the part was considered uniform to simplify the construction of mathematical and physical models.

In this study, we will investigate the thermal field of the casting during solidification in much greater depth. Because for casting technologies, in addition to the liquidus and solidus temperatures, two more “starting points” are important.

**Figure 1:**
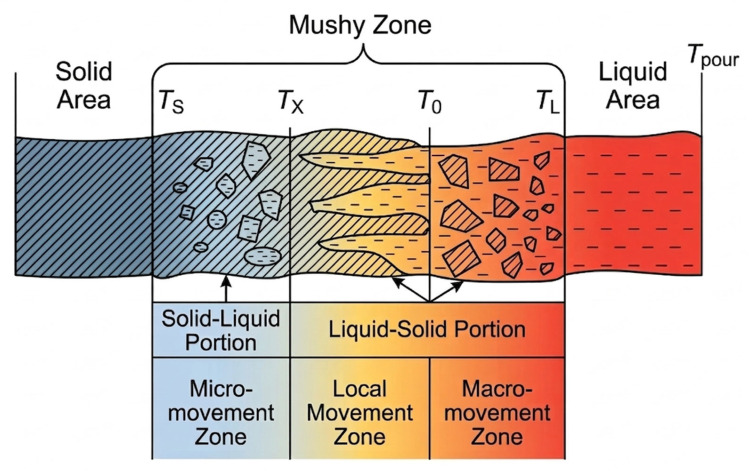
Structure of the mushy zones: *T_S_*—solidus temperature; *T_X_*—feeding temperature; *T*_0_—zero fluidity temperature; *T_L_*—liquidus temperature.

Casting alloys in most cases solidify in the temperature range, and the solidification process is sequential from the surface of the casting to its center [59,60,61]. As a result, the solidification front is not flat, but has a certain thickness, which depends on the solidification interval and the kinetics of temperature changes across the casting cross section [39]. In the solidified casting, three zones can be distinguished: solid, solidification, and liquid. They are divided by the solidus and liquidus temperatures (Figure 1).

In accordance with the solid, solidification, and liquid regions, three displacement zones can be identified [41,42]. In the macroscopic displacement zone, solid crystals float freely in the liquid and are removed along with the fluid during the pouring out of the liquid residue. This zone is separated from the local displacement zone by the zero fluidity temperature *T*_0_.

In the zone of local liquid phase displacements, the crystals form the coherent volumetric framework, yet the liquid moves relatively freely between them. Should cavities arise due to metal shrinkage during solidification, the liquid can fill them, thereby ‘feeding’ the casting and ensuring the dense, non-porous structure. This critical process is implemented in practice by attaching risers to the casting for its feeding.

In the microscopic displacement zone, the liquid phase is divided by solid-phase crystals into isolated micro-volumes, and liquid movement occurs only within these micro-volumes. Since they are isolated, no feeding occurs during their solidification, which leads to the formation of micro-shrinkage porosity. The feeding temperature boundary, T_X_, separates the zones of microscopic and local displacements (Figure 1).

Establishing the solidification dynamics of the casting while tracking the progression of the *T_L_*, *T*_0_, *T_X_*, and *T_S_* temperature fronts remains difficult from both a mathematical and a physical perspective. Although modern numerical tools are widely used for solidification analysis, the reliable representation of these fronts still depends on the quality of boundary-condition identification, interfacial heat-transfer modeling, and thermophysical input data. In this context, an analytical formulation remains valuable as a physically interpretable basis for subsequent numerical refinement.

The object of the study is a casting made of Al–5%wt.Cu aerospace-grade aluminum alloy, represented by a solid cylinder 20 mm in diameter and 200 mm in length. The manufacturing of this part is implemented in both expendable (sand–clay) and reusable (steel) molds. The subject of the study is the distribution of thermal fields within this part during its solidification process.

The aim of this study is to develop an analytical calculation methodology that enables obtaining the distribution of thermal fields in aluminum alloy castings during the solidification process in sand and metal molds.

## 3. Model Description

**Figure 2:**
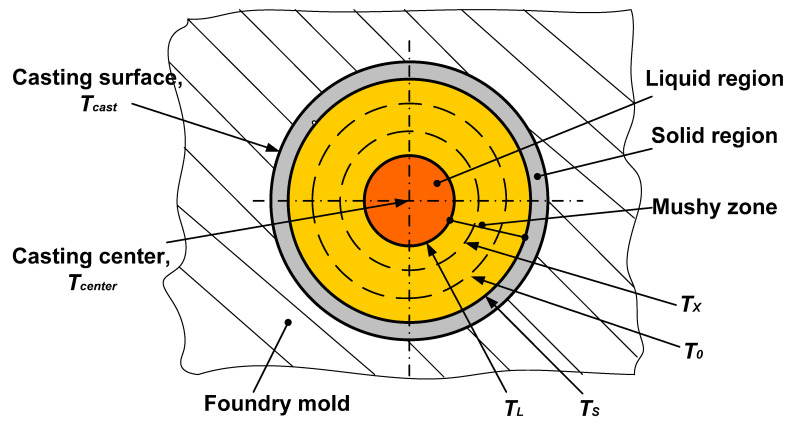
Calculation scheme for determining the temperature fields of the casting.

The object of the calculation is a cylindrical casting of Al–5%wt.Cu alloy with a diameter of 20 mm (Figure 2). The material of the casting mold: sand–clay or metal (steel).

The following initial data (Table 1) were used for calculations [14,62].

All quantitative results presented below correspond to the thermophysical constants and process temperatures adopted in Table 1. The proposed analytical procedure is deterministic with respect to these input data; therefore, any change in the pouring temperature, the initial mold temperature, the characteristic phase-transformation temperatures, or the effective thermophysical and interfacial parameters will lead to corresponding changes in the calculated time and temperature characteristics. This is especially important for sand molds, whose effective thermal response depends on binder content, moisture, and degree of compaction. Accordingly, the numerical results presented in this paper should be interpreted as specific predictions for the adopted “alloy–mold” system, whereas the broader contribution of the study lies in the analytical procedure itself for reconstructing the temperature field.

The analytical methodology is based on the construction of the non-stationary heat-transfer model for the casting–mold system during the solidification of the cylindrical Al–5 wt.% Cu casting in sand and metal molds. In contrast to approaches that rely primarily on integral estimates of solidification time or simplified static boundary conditions, the methodology proposed by us is aimed at reconstructing the non-stationary thermal field, in particular the temperature of the casting surface, its thermal center, and the solidification front advancing over time.

The general methodology for calculating the solidification process of the casting in the sand mold is presented in the following sequence:

- The total solidification time of the casting was established. For this purpose, a well-known set of analytical relationships was used; since these are not the original findings of the authors, they are not presented in the Analytical Solution section. Specifically, G. Balandin’s universal formula was applied.
- The duration of superheat removal was determined, i.e., the time required for the casting surface to cool to the liquidus temperature. A known but less common formula was used for this purpose, which is provided as Equation (1).
- The duration for the casting surface to cool to the solidus temperature was established using the calculation method developed by the authors. All boundary conditions and the solution to this problem are presented as Equations (2)–(4), along with corresponding explanations.
- Numerical values for the zero-fluidity temperature *T*_0_ and the feeding temperature T_X_ were calculated. Using the developed Equation (4), the time required for the casting surface to cool to each of these temperatures was determined.
- The configuration features of the casting were taken into account. Its cylindrical shape causes the non-linear advancement of the solidification front from the surface to the center. The authors developed the calculation method that accounts for the casting geometry. The results of the solidification front propagation, determined by Equations (5) and (6), are presented in Table 2.
- The temperature distribution in the solidified and liquid parts of the casting was determined. The calculation method developed by the authors was used, which considers the volume ratio of the solidified and liquid regions, thereby accounting for the casting configuration. The calculation was performed using Equation (8), and the results are shown in Table 3.
- The temperature distribution across the casting cross-section during its total solidification period was determined, and the temperature gradient between the surface and the center was calculated. Such a calculation using this integrated methodology is presented for the first time.

**Table 2:** Distribution of solid and liquid layers in the casting of Al–5%wt.Cu alloy with the diameter of 20 mm, poured into the sand mold.

	τ, *s*	15 (τ*_over_*)	19 (τ_0_)	22 (τ*_x_*)	27 (τ*_S_*)	50	75	100	125	150	175	190 (τ_T_)
1	δ*_NT_*, *m*	0	0	0	0	0.0019	0.0027	0.0033	0.0039	0.0043	0.0047	0.0050
2	δ*_NT_*/*R_C_*	0	0	0	0	0.374	0.540	0.666	0.772	0.865	0.949	1.000
3	*V_S_*	0	0	0	0	0.000117	0.000170	0.000209	0.000242	0.000272	0.000298	0.000314
4	*d*, *m*	0	0	0	0	0.0158	0.0135	0.0116	0.0096	0.0073	0.0045	0
5	δ*_S_*, *m*	0	0	0	0	0.0021	0.0032	0.0042	0.0052	0.0064	0.0078	0.0100

**Table 3:** Calculation of the temperature field of the casting made of Al–5%wt.Cu alloy with the diameter of 20 mm, poured into the sand mold.

τ, *s*	15 (τ*_over_*)	19 (τ_0_)	22 (τ*_x_*)	27 (τ*_S_*)	50	75	100	125	150	175	190 (τ_T_)
*T_center_*, K	1040	1015	1005	975	910	865	835	808	796	784	780
*T_cast_*, K	910	867	833	780	743	733	728	724	721	718	715

An important feature of the approach is that we treat the cooling rate of the casting surface as a quantity that varies with time. This makes it possible to derive an analytical differential equation describing the evolution of the surface temperature, instead of prescribing it in advance. From a methodological standpoint, this reduces the uncertainty associated with the specification of boundary conditions and provides a more physically grounded basis for reconstructing the thermal field.

Another important feature of the methodology is the consideration of the object geometry. For the cylindrical casting, the advancement of the solidification front cannot be adequately described solely by the classical linear square root law. Therefore, we supplemented the calculation scheme with the volumetric interpretation of the phase transformation, within which the ratio of the solid and liquid regions is determined by their volumes rather than only by the nominal thickness of the solidified layer. This approach is particularly appropriate for bodies of cylindrical shape and improves the physical consistency of the analytical description of front propagation and temperature redistribution during solidification.

The methodology also goes beyond the traditional use of only the liquidus and solidus temperatures. In addition to $T_{L}$ and $T_{S}$, we included in the analytical scheme the zero-fluidity temperature $T_{0}$ and the feeding temperature $T_{X}$, which are of important technological significance for interpreting the transition from freely flowing melt to conditions of restricted feeding. Owing to this, the model is intended not only to describe heat transfer itself, but also to identify temperature intervals associated with the possible formation of micro shrinkage porosity. In this way, the methodology proposed by us more closely links thermal-field analysis with the technological assessment of casting quality.

For metal molds, we apply the inverse analytical sequence. The general methodology for the calculations of the casting solidification process in a metal mold is presented in the following sequence:

- The total solidification time of the casting was established. For this purpose, the well-known set of analytical relationships was used. Since these are not the original findings of the authors, they are not presented in the Analytical Solution section. Specifically, A. Veinik’s formula was applied.
- The duration for the casting center to cool to the liquidus temperature was determined. The known but less common formula was used for this purpose, presented as Equation (9), with additional calculations performed using Equations (10)–(12).
- The casting surface temperature corresponding to the moment the center reaches the liquidus temperature was established. For this, the authors applied Equation (13), which had not been previously used exclusively for “reverse calculation” (determining center temperature based on surface temperature).
- The dynamics of the casting surface cooling were established. The calculation method developed by the authors, previously described for the sand mold, was applied. For the metal mold, the cooling equation is designated as (14).
- Using Equation (14), the duration for the casting surface to cool to the zero-fluidity temperature *T*_0_ and the feeding temperature *T_X_* was calculated.
- The dynamics of the solidification front advancement from the surface to the center were calculated. The methodology is analogous to the one presented for the sand mold, meaning the cylindrical configuration of the casting was also taken into account. The results are presented in Table 4.
- The temperature distribution in the solidified and liquid parts of the casting was determined, also considering its shape. The results are shown in Table 5.
- The temperature distribution across the casting cross-section during the total solidification period was determined. The temperature gradient between the surface and the center was calculated.
- The potential features of structure formation and the development of casting properties in sand and metal molds were predicted. These features, driven by the solidification rate and the temperature gradient across the cross-section, were analyzed, and the theoretical comparison between the two mold types is provided.

**Table 4:** Distribution of solid and liquid layers in the casting of Al–5%wt.Cu alloy with the diameter of 20 mm, poured into the steel mold.

	τ, *s*	2	4	6	8	10	12	14	16 (τ_T_)
1	δ*_NT_*, *m*	0	0	0	0.0022	0.0032	0.0039	0.0045	0.0050
2	δ*_NT_*_/_*R_C_*	0	0	0	0.450	0.632	0.780	0.840	1.000
3	*V_S_*	0	0	0	0.000140	0.000198	0.000245	0.000281	0.000314
4	*d*, *m*	0	0	0	0.015	0.012	0.009	0.006	0
5	δ*_S_*, *m*	0	0	0	0.0026	0.0040	0.0053	0.0070	0.0100

**Table 5:** Calculation of the temperature field of the casting made of Al–5%wt.Cu alloy with the diameter of 20 mm, poured into the steel mold.

τ, *s*	2.0	3.1	4.0	4.1	4.7	6.0	8.0	10.0	12.0	14.0	16.0
*T_center_*, K	990	955	918	910	895	870	840	818	800	787	780
*T_cast_*, K	975	910	867	860	833	780	757	749	745	741	738

Instead of starting from the surface temperature, we first determine, using the available analytical relationships, the variation in temperature in the thermal center of the casting, and then, on this basis, reconstruct the thermal state of its surface. This inversion step is one of the most distinctive methodological features of the study, since it makes it possible to avoid the direct use of mutually dependent quantities during the reconstruction of the thermal field and forms the more transparent analytical algorithm for permanent-mold casting conditions.

For the convenience of reproducing the methodology, all derived mathematical relationships and the final working equations are presented in the Results section directly in the context of their application to sand and metal molds.

## 4. Analytical Solution

### 4.1. Calculation of Thermal Fields in the Aluminum Alloy Casting During Solidification in the Sand Mold

The total solidification time for an Al–5%wt.Cu alloy casting with the diameter of 20 mm in the sand mold was calculated using established equations summarized in [14], resulting in the value of 190 s.

To derive the analytical equation for the casting’s surface temperature variation, reliable data regarding its values at minimum two distinct points in time are required. These points include the initial moment (mold pouring, when the metal is at 1123 K) and the moment when superheat dissipation is complete (when the casting surface cools to the liquidus temperature of 910 K, marking the onset of solidification). For this purpose, the equation for calculating the time of superheat dissipation from [14] was employed:

(1) $$\tau_{\mathrm{over}}={\left[\frac{C_{L}\cdot \rho_{L}\cdot R_{C}\cdot (T_{\mathrm{pour}}-T_{L})}{1.128\cdot b_{M}\cdot (T_{\mathrm{pour}}-T_{M})}\right]}^{2},$$

where *C_L_* is the heat capacity of the alloy in the liquid state, J/(kg·K); *ρ_L_* is the density of the alloy in the liquid state, kg/m^3^; *R_C_* is the combined size of the casting, m; *T_pour_, T_L_, T_M_* are the pouring temperatures of the alloy, liquidus, mold, K; *b_M_* is the heat accumulation coefficient of the casting mold W·s^1/2^/(m^2^·K).

At the moment *τ = τ*_over_ directly on the surface of the casting the temperature value is equal to *T_L_* = 910 K.

It is incorrect to use Equation (1) to determine the time of cooling of the casting below the liquidus temperature, because in the volume of the casting from the moment *τ = τ*_over_ in addition to the temperature decrease, the latent heat of solidification begins to be released, which the equation does not take into account.

To determine the time of cooling of the surface of the casting to the solidus temperature (780 K), we used the method presented by us in the previous publication [58]. The formulation of this problem is as follows:

- Initial temperature *T*_pour_ = 1123 K;
- The temperature of the surface of the casting at the moment of time is equal to *T_L_* = 910 K;
- Initial temperature of the mold *T_M_* = 293 K;
- Solidus temperature *T_S_* = 780 K.

The cooling rate v for such the process is the variable quantity and is described by the differential equation: $v=\frac{d(T-T_{M})}{d\tau }$.

Then

(2) $$\frac{\mathrm{dT}}{d\tau }=k\cdot (T-T_{M}),$$

where T is the surface temperature of the casting; *T_M_* is the mold temperature; *τ* is time.

Hence:

(3) $$\frac{\mathrm{dT}}{T-T_{M}}=k\cdot d\tau ;\ln\left(T-T_{M}\right)=k\cdot \tau +\ln C;\frac{\ln(T-T_{M})}{C}=k\cdot \tau ;$$

The analytical law of cooling the casting surface has the form:

$$T-T_{M}=C\cdot e^{k\cdot \tau }.$$

To establish the constant C, we apply the first boundary condition: *T*_τ = 0_ = *T*_pour_ = 1123 K; *T_M_* = 293 K. Therefore:

1123−293=C⋅1; *C* = 830.

Then Equation (3) takes the form:

$$T-293=830\cdot e^{k\cdot \tau }.$$

To establish the coefficient k, we apply the second boundary condition:

*T*_τ=15s_ = *T_L_* = 910 K. Therefore:

$$910-293=830\cdot e^{k\cdot 15}.$$

$$e^{15\cdot k}=0.743.$$

$$e^{k}=(0.743{)}^{\frac{1}{15}}=0.743^{0.067}.$$

Thus, the mathematical law of cooling the casting surface has the final form:

(4) $$T-293=830\cdot 0.743^{0.067\cdot \tau }.$$

where *τ* is time, s; *T* is the temperature of the casting surface at the calculated time, K.

Using Equation (4), we find the time of cooling of the casting surface to the solidus temperature (780 K):

$$780-293=830\cdot 0.743^{0.067\cdot \tau }.$$

$$\ln\left(780-293\right)=\ln830+0.067\cdot \tau \cdot \ln0.743;\;\;6.19=6.72-0.297\cdot 0.067\cdot \tau ;$$

$$\tau =\frac{6.72-6.19}{0.0199}=27\;s.$$

As the result of the calculations, the following was established:

- At the moment *τ* = 0 at all points (on the surface of the casting and in its center, the temperature *T*_pour_ = 1123 K;
- At the moment on the surface of the casting, the temperature *T_L_* = 910 K;
- At the moment on the surface of the casting, the temperature *T_S_* = 780 K;
- Equation (4) allows you to determine the temperature of the surface of the casting at any time in the period from 0 to 27 s. Thus, it is possible to calculate the time of cooling of the surface of the casting to temperatures *T*_0_ (zero fluidity temperature) and *T_X_* (feeding temperature).

We determine the time of cooling to the zero fluidity temperature, which is 867 K:

$$867-293=830\cdot 0.743^{0.067\cdot \tau }.$$

ln574=ln830+0.067⋅τ⋅ln0.743

$$\tau_{0}=\frac{6.72-6.35}{0.067\cdot 0.297}=19\;s.$$

We determine the time of cooling to the feeding temperature, which is approximately 833 K:

$$833-293=830\cdot 0.743^{0.067\cdot \tau }.$$

ln540=ln830+0.067⋅τ⋅ln0.743

$$\tau_{x}=\frac{6.72-6.29}{0.067\cdot 0.297}=22\;s.$$

The increase in the solidified layer thickness on the surface of the casting occurs from the moment the solidus temperature is reached on the surface (*τ_S_* = 27 s) until the moment of complete solidification *τ_T_* = 190 s. That is, the solid zone of the casting gradually moves from its surface to the center during the time τ = 190 − 27 = 163 s.

If the casting were flat, the absolute thickness of the solidified layer would increase in exact accordance with the “square root” law. But the casting is cylindrical, so its shape must be taken into account.

Since the solidification process is characterized by the amount of heat released per unit volume, the calculation of the advance of the solidification front should be attributed not to the thickness of the solidified layer, but to its volume.

The nominal thickness of the solidified layer, which for the flat casting is equal to the real thickness, can be determined by the equation:

(5) $$\delta_{\mathrm{NT}}=K_{X}\cdot \sqrt{(\tau -\tau_{S}),}$$

where *δ*_NT_ is the conditional thickness of the solidified layer, m; *K_X_* is the coefficient of the speed of the solidification front, m/s^1/2^; τ is the calculated time, s; *τ_S_* is the time of cooling the casting surface to the solidus temperature, s.

To find the *K_X_* coefficient, we use Equation (5), taking *δ*_NT_ = *R_C_* (the reduced size or cooling modulus of the casting) and τ = τ_T_ (the time of complete solidification):

$$K_{X}=\frac{Rc}{\sqrt{\tau_{T}-\tau_{S}}}=\frac{0.005}{\sqrt{190-27}}=0.00039\;\frac{m}{s^{1/2}}.$$

To take into account the cylindrical shape of the casting, the calculations were performed in the following sequence:

1. The estimated volume of the casting in conventional units is determined. The estimated volume of the casting is (according to its dimensions): $V=\frac{\pi \cdot D^{2}}{4}=\frac{3.14\cdot 0.02^{2}}{4}=0.000314$.
2. The conditional thickness of the solidified layer δ is determine *δ_NT_* for the number of consecutive time points (Table 2, line 1) according to Equation (5). The calculated data are given in Table 2, line 2. For this purpose, the value of the coefficient of the speed of advance of the solidification front and Equation (5) were used, in which specific values of time points were substituted for τ.
3. The relative thickness of the solidified layer δ is determine δ*_NT_*/*R_C_* for each time point, the results are given in Table 2, line 3.
4. The relative volume of the solidified layer is determined *V_S_* for each point in time (Table 2, line 4).
5. The diameter of the unsolidified metal zone was determined (Table 2, line 5). For this, the geometric equation was used:

(6) $$d=\sqrt{D^{2}-\frac{4\cdot V_{S}}{\pi }},$$

where D is the outer diameter of the casting (*D* = 0.02 m).

6 The actual thickness of the solidified layer for each time point *δ_S_* was calculated (Table 2, line 5).

It is a real possibility to calculate the change in the temperature of the casting surface at the stage from T_pour_ to T*_S_* according to the derived Equation (4). Below the solidus temperature T*_S_,* this equation loses its validity, since the solidification process occurs with the release of latent heat of solidification, which is not taken into account here, and the transition from the liquid to the solid state leads to the cardinal change in the thermophysical characteristics of the metal. Therefore, as shown in our previous work [58], the casting at this stage is considered as the two-layer structure. The surface layer is already solid, and its thickness gradually increases (according to Table 2); the rest of the metal is the conditionally liquid phase. Accordingly, the solid and liquid phases have different thermophysical characteristics.

**Figure 3:**
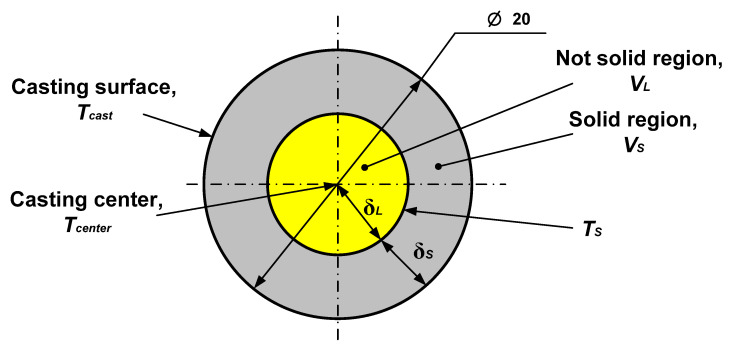
Calculation scheme for determining the dynamics of the solidification front advancement in the casting (the values of V_S_ at different times are given in Table 2).

The calculation scheme is given in Figure 3.

The task of distributing temperature fields in such system is to establish the following initial conditions:

1. Dynamics of changes in the temperature of the center of the casting.
2. Dynamics of the solidification front advancement from the surface to the center of the casting.
3. Dynamics of changes in the temperature of the casting surface.

Condition No. 2 is the solved issue that has already been presented in Table 2. As for the change in surface temperature (condition No. 3), it is possible to calculate it only at the initial stage (cooling to the solidus temperature *T_S_*). The dynamics of the temperature change in the center of the casting can also be established using the known initial (1123 K) and final values at the moment of completion of solidification (780 K), for which the calculation mechanism in the form of Equations (2)–(4) can be used.

Now, at fixed points in time, the temperature values in the center of the casting and the position of the solidification front are known. By conditionally dividing the casting into liquid and solid parts and considering it as the two-layer wall (see Figure 3), it would be correct to assume that the heat fluxes in these parts will be equal, as was done in [58] $q_{S}=q_{L}$, and therefore:

(7) $$\frac{\lambda_{L}}{\delta_{L}}\cdot (T_{\mathrm{center}}-T_{S})=\frac{\lambda_{S}}{\delta_{S}}\cdot (T_{S}-T),$$

where *λ_L_* and *λ_S_* are thermal conductivity coefficients of the alloy in the liquid and solid states, W/(m·K); *δ_L_* and *δ_S_* are thickness of liquid and solidified layer, m; *T*_center_ is temperature in the center of the casting, K; *T_S_* is solidus temperature, K; and *T*_cast_ is casting surface temperature, K.

From this equation, the surface temperature of the casting is:

$$T_{\mathrm{cast}}=T_{S}-\frac{\lambda_{L}\cdot \delta_{S}}{\delta_{L}\cdot \lambda_{S}}\cdot (T_{\mathrm{center}}-T_{S})$$

To take into account the cylindricity of the casting, instead of the ratio of the thickness of the solid and liquid layers, it is necessary to take into account the ratio of their volumes:

(8) $$T_{\mathrm{cast}}=T_{S}-\frac{\lambda_{L}\cdot V_{S}}{V_{L}\cdot \lambda_{S}}\cdot (T_{\mathrm{center}}-T_{S})$$

where *V_L_* and *V_S_* are the volumes of the liquid and solid parts of the casting, respectively.

Based on the previously calculated values of the volumes of the solid and liquid layers at different times (see Table 2), the surface temperature was calculated. For example, at time τ = 50 s:

$$T_{\mathrm{cast}}=780-\frac{102\cdot 0.000117}{(0.000314-0.000117)\cdot 215}\cdot (910-780)=743\;K.$$

**Figure 4:**
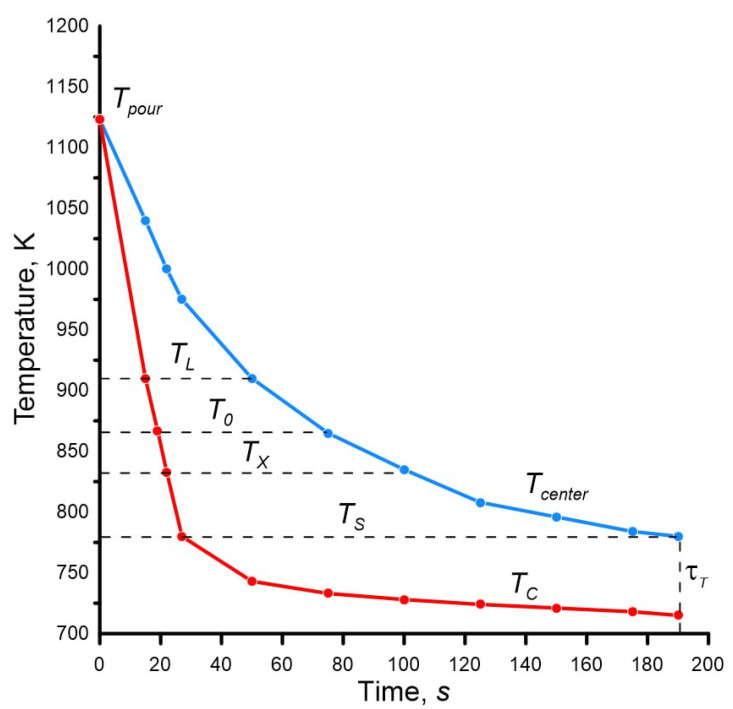
Temperature distribution in the aluminum alloy casting (see Figure 2) poured into the sand mold, during the period from pouring to complete solidification (the blue line is the temperature change in the center, the red line is the temperature change on the surface).

The calculation results are given in Table 3, and the calculated temperature field of the casting is given in Figure 4.

From Table 3 and Figure 4, it is clear that the temperature difference in the casting is quite significant and is especially evident at the initial stage after pouring. The reason is the relatively rapid cooling of the surface of the casting, which is in contact with the mold, compared to the center of the casting.

After the casting surface begins to solidify (cooling below the temperature *T_S_*), the temperature difference across the casting cross-section decreases during the solidification process. However, during the solidification process, the temperature difference is large and has the significant impact on the heat transfer processes in the casting–mold system and inside the casting itself.

After cooling the center of the casting to temperature *T_X_* in casting, the solid–liquid zone remains, during the final solidification of which feeding does not occur, which is the main reason for the appearance of shrinkage porosity in the inner layers of castings.

### 4.2. Calculation of Thermal Fields of the Aluminum Alloy Casting During Its Solidification in the Metal Mold

To determine the time of solidification of castings in metal molds, there are known equations, which are also collected in professional publications, for example [14]. Regarding the determination of the cooling dynamics of different parts of the casting, the available equations are fragmentary and do not allow calculating the real thermal field of the casting.

The time of solidification of the cylindrical casting of Al–5%wt.Cu alloy in the steel mold, using the generally accepted calculation method, is 16 s.

The remaining equations for determining thermal parameters are much less widespread and can be found in the limited number of, mostly old, publications. Therefore, we consider it necessary to present the found equations in full.

It is recommended to determine the time of heat removal from overheating in metal molds as follows:

(9) $$\tau_{R}=\frac{C_{L}\cdot \rho_{L}\cdot R_{C}}{\alpha_{01}}\cdot \ln\frac{T_{\mathrm{pour}}-T_{1}}{T_{L}-T_{1}}$$

where *C_L_* is specific heat capacity of the alloy in the liquid state; *ρ_L_* is density of the alloy in the liquid state; *R_C_* is the reduced size of the casting; *α*_01_ is heat transfer coefficient from the casting to the mold; *T*_pour_ is pouring temperature; *T_L_* is liquidus temperature; and *T*_1_ is calorimetric temperature.

Some quantities from Equation (9) require additional calculations. To determine the calorimetric temperature, the equation is used:

(10) $$T_{1}=\frac{T_{pour}+\frac{C_{M}\cdot \rho_{M}\cdot \delta_{M}}{C_{L}\cdot \rho_{L}\cdot \delta_{C}}\cdot T_{M}}{1+\frac{C_{M}\cdot \rho_{M}\cdot \delta_{M}}{C_{L}\cdot \rho_{L}\cdot \delta_{C}}}$$

where *C_M_* is specific heat capacity of the mold material; *C_L_* is specific heat capacity of the alloy in the liquid state; *ρ_M_* is density of the mold material; *ρ_L_* is density of the alloy in the liquid state; *T_pour_* is pouring temperature; *T_M_* is initial mold temperature; and $\;\frac{\delta_{M}}{\delta_{C}}$ is the ratio of the relative thicknesses of the mold and the casting. For the cylindrical casting, the ratio of relative thicknesses is taken as:

(11) $$\frac{\delta_{M}}{\delta_{C}}=2\cdot \frac{l_{M}}{R}+(\frac{l_{M}}{R}{)}^{2}$$

where *l_M_* is the actual wall thickness of the metal mold; *R* is the radius of the cylindrical casting.

The heat transfer coefficient from the casting to the mold is determined by the equation:

(12) $$\alpha_{01}=\frac{\lambda_{0}}{\delta_{0}}\cdot \frac{T_{\mathrm{pour}}-T_{M}}{T_{\mathrm{pour}}-T_{1}},$$

where *δ*_0_ is the thickness of the protective coating layer on the mold surface; *ƛ*_0_ is the thermal conductivity coefficient of the coating on the mold surface; *T_pour_* is pouring temperature; *T_M_* is initial temperature of the mold; *T*_1_ is calorimetric temperature.

According to Equation (12), taking into account that *l_M_* = 0.03 m, and *R* = 0.01 m, we obtain:

$$\frac{\delta_{M}}{\delta_{C}}=2\cdot \frac{0.03}{0.01}+(\frac{0.03}{0.01}{)}^{2}=15.$$

According to Equation (10), the calorimetric temperature is:

$$T_{1}=\frac{1123+\frac{750\cdot 7500}{1200\cdot 2700}\cdot 15\cdot 523}{1+\frac{750\cdot 7500}{1200\cdot 2700}\cdot 15}=546\;K.$$

According to Equation (12), the heat transfer coefficient provided that the protective coating is based on powdered quartz with the thickness of 0.2 mm with the thermal conductivity coefficient *λ*_0_ = 0.350 W/(m·K):

$$\alpha_{01}=\frac{0.350}{0.0002}\cdot \frac{1123-523}{1123-546}=1820\frac{W}{m^{2}\cdot K}.$$

The time of heat removal from overheating according to Equation (9) is:

$$\tau_{R}=\frac{1200\cdot 2700\cdot 0.005}{1820}\cdot \ln\frac{1123-546}{910-546}=4.1\;s.$$

In the metal mold, the cooling dynamics of the casting are significantly different from those in the sand mold. First of all, after the melt comes into contact with the surface of the metal mold, very fast formation of the solidified crust. Therefore, it is obvious that the calculated time withdrawal heat of superheating, which is 0.25 of the total time of the casting solidification, refers to the thermal center of the casting, not to its surface. Thus, it is absolutely correct to state that at time *τ*_R_ = 4.1 s in the center of the casting, the temperature is equal to the liquidus point (*T_center_ = T_L_*).

The complexity of the analytical calculation of the thermal field of the casting, compared to the sand mold, lies in the fact that, in the previous case, it was possible to analytically calculate the dynamics of changes in the surface and center temperatures of the casting. In the metal mold, equations exist for calculating the surface temperature, but none of them take into account the time parameter; that is, they do not allow for determining the main thing—the dynamics of changes in this temperature. Therefore, the dynamics of the distribution of thermal fields across the cross-section of the casting remains an urgent scientific issue.

To calculate the dynamics of surface temperature changes, you need to construct an equation, similar to (4). But for this, you need to know the exact values of this temperature to a lesser extent at two different points in time.

For this at the moment $\tau_{R}=4.1\;s$, we will use a previously existing equation:

(13) $$\frac{T_{\mathrm{Me}}-T_{M}}{T_{C\mathrm{AST}}-T_{M}}=1+\frac{b_{M}}{b_{S}}\cdot \mathrm{erf}\frac{y}{2\cdot \sqrt{a_{L}\cdot \tau }},$$

where *T_Me_* is temperature at the casting point at the distance y from the surface; *T_M_* is initial mold temperature; *T*_CAST_ is casting surface temperature; *b_M_* and *b_S_* are coefficients of heat accumulation, respectively, of the metal mold and the alloy of the casting in the solid state; *a_L_* is thermal diffusivity coefficient of the alloy in the liquid state; and τ is time.

The authors proposed this equation to determine the temperature of the casting surface, and then from it to calculate the thermal field *T_Me_* at different points. However, to determine the surface temperature *T_CAST_,* obviously the value of *T_Me_* is required, so this is a typical example of using interdependent quantities in calculations, which makes the results unreliable.

In our case, the temperature in the center of the casting was determined by direct calculation, and therefore it is reliably known that at the point in time $\tau_{R}=4.1\;s$ *T_Me_ = T_CENTER_* = 910 K.

Then, taking into account that the parameter y from Equation (13) is the distance from the surface to the thermal center of the casting and is equal to its reduced size *y* = *R_WITH_* = 0.005 m, we obtain the value of the surface temperature at this moment:

$$T_{C\mathrm{AST}}=\frac{T_{\mathrm{center}}+T_{M}\cdot \frac{b_{M}}{b_{S}}\cdot \mathrm{erf}\frac{y}{2\sqrt{a_{L}\cdot \tau }}}{1+\frac{b_{M}}{b_{S}}\cdot \mathrm{erf}\frac{y}{2\sqrt{a_{L}\cdot \tau }}}=\frac{910+523\cdot \frac{17,540}{26,000}\cdot \mathrm{erf}0.220}{1+\frac{17,540}{26,000}\cdot \mathrm{erf}0.220}=856\;K.$$

The equation for cooling the casting surface will have the form similar to (4), and the method of obtaining it is similar to the calculations using Equations (2) and (3).

(14) $$T_{C\mathrm{AST}}-523=600\cdot 0.555^{0.24\cdot \tau }$$

Equation (14) determines the time of cooling the casting surface to temperatures:

- Liquidus (*T_L_*)—3.1 s;
- Zero fluidity (*T*_0_)—4.0 s;
- Feeding (*T_X_*)—4.7 s;
- Solidus (*T_S_*)—6.0 s.

Considering that the process of solid layer growth on the surface of the casting begins from the moment the temperature *T_S_* is reached there, i.e., 6.0 s, and ends in the center of the casting at 16.0 s, the coefficient of the rate of advance of the solidification front from Equation (5):

$$K_{X}=\frac{R_{C}}{\sqrt{\tau_{T}-\tau_{S}}}=\frac{0.005}{\sqrt{16-6}}=0.00158\;\frac{m}{s^{1/2}}.$$

The calculation of the thermal field of an aluminum casting, which is solidified in the metal mold, must also take into account its cylindrical shape. For this, the method similar to the calculations for the sand mold was used (Equations (5) and (6)). The results of the calculation of the casting solidification dynamics are given in Table 4. The physical content of all the given quantities is similar to the previous calculation.

After that, we determine the temperature fields of this casting.

**Figure 5:**
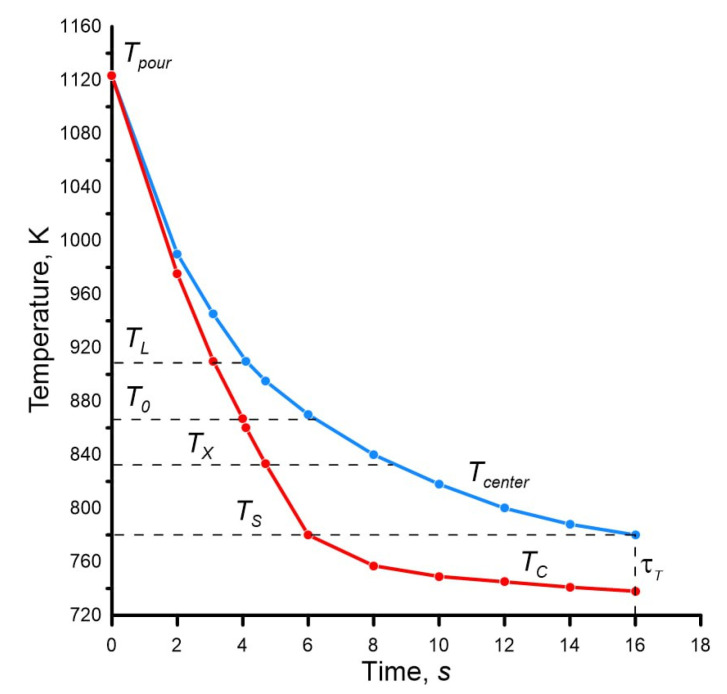
Temperature distribution in an aluminum alloy casting (see Figure 2) poured into the metal mold during the period from pouring to complete solidification (the blue line is the temperature change in the center, the red line is the temperature change on the surface).

The calculation results are given in Table 4, and the calculated temperature field of the casting is shown in Figure 5.

To determine the change in surface temperature after cooling to the solidus temperature, the previously presented method used Equation (8).

The rapid dynamics of solidification of the casting in the metal mold results in the smaller temperature difference between the surface and the center, which has a positive effect on structure formation and significantly reduces the likelihood of shrinkage defects.

**Figure 6:**
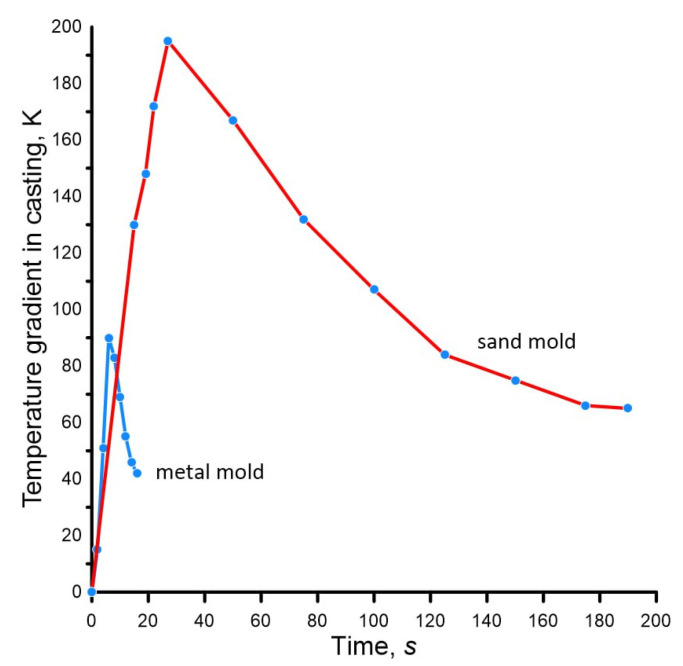
Dynamics of change in time of the temperature difference between the center and the surface of an aluminum alloy casting (see Figure 2) depending on the mold material (sand and metal).

The comparative dynamics of changes in the temperature field of the casting in the sand and metal mold is shown in Figure 6.

The temperature difference between the center and the surface of the casting, firstly, reaches quite large values, given the casting diameter of 20 mm, and secondly, it is much larger in the sand mold. Uneven cooling of different layers of the casting leads to the formation of the non-uniform structure, and is also the cause of the formation of casting defects.

The dynamics of the temperature difference between the surface and the center of the casting also has its own explanation. At the initial stage, a rapid increase is observed. As the result, in the sand mold, the temperature difference in the casting reaches 195 K, in the metal mold—90 K. In both cases, the maximum occurs at the moment when the temperature on the surface of the casting is equal to the solidus point T_S_. After this, a gradual decrease in the temperature difference is observed due to the combination of two physical phenomena: the release of latent heat of solidification on the surface of the casting and heat transfer from the inner to the outer layers of this same heat of solidification. This causes a significant slowdown in the cooling of the surface of the casting (see Figure 4 and Figure 5), and to a lesser extent—the center of the casting (see Figure 4 and Figure 5) and causes a decrease in the temperature difference.

## 5. Discussion of Results

The presented results represent the combination of previously known methods for calculating temperature fields in cast parts and those created by the authors of this article. The main goal has been achieved: the temperature fields have been analytically calculated using the example of the casting made of Al–5%wt.Cu alloy during its solidification in sand (sand–clay) and metal (steel) molds.

Variants of such calculations, presented in literary sources from about 1960 to the present, are quite numerous. But the issue does not lose its relevance, as evidenced by the emergence of new equations, analytical equations and, of course, computer modeling methods. The authors consider the main shortcomings of the existing calculations and, accordingly, computer algorithms based on them to be the lack of attention to the dynamics of temperature changes in surfaces and thermal centers of cast parts, the lack of consideration of the dynamics of the solidification front advance, and therefore the distribution of temperature fields in castings always contains a certain kind of inaccuracy.

The authors’ achievements include new solutions:

1. Analytical calculation of the cooling dynamics of the surface temperature of the casting in contact with the sand mold during the entire period from pouring to complete solidification. As can be seen from Table 2 and Table 3, as well as Figure 4 and Figure 5, the final temperature is lower than the solidus point, which none of the previous methods made it possible to obtain.
2. Analytical calculation of the cooling dynamics of the casting surface in contact with the metal mold. What is new is that this temperature is calculated depending on the values of the temperature of the casting center, which are determined by previously available equations. But such “reverse” calculation is performed for the first time.
3. Taking into account, in addition to the liquidus temperatures *T_L_* and solidus *T_S_*, such important points from the point of view of pouring and solidification as the temperature of zero fluidity *T*_0_ and the feeding *T_X_*. In Figure 4 and Figure 5, you can get information about the position of all the considered temperatures at any time. For the Al—5% Cu alloy, which has the solidification temperature interval of 130 K, the temperature distribution is extremely important, especially in the zones limited by these points, since this directly affects the formation of shrinkage porosity, and as the consequence, the density, hydrodensity, and mechanical properties of the metal.
4. Establishing the complete picture of temperature fields using the example of the cylindrical casting with the diameter of 20 mm in sand and metal molds solely on the basis of analytical calculations.

It should be noted that the geometric factor was taken into account, since it is known that the solidification front and thermal fields in the flat and cylindrical casting are distributed differently. Therefore, as shown in Figure 3, it was not the linear ratios of the solid and liquid zones that were taken into account, but the volumetric ones.

The article provides the detailed algorithm of analytical solutions that can be repeated for other casting designs, mold materials, etc. The purpose of such a detailed presentation: the ability to reproduce similar calculations on established examples. The presented equations are not only the authors’ own developments, as indicated by the relevant references to the original sources. Some of the equations previously presented by the authors in another publication are also used here. The fundamental difference is that previous work was devoted to the temperature field of the hollow steel cast part with an internal core, so these calculations have little in common.

It is planned to finally confirm the reliability of the presented methods in the future with real physical experiments, after which we will proceed to building computer models, or better yet, to refining the existing computer models of leading manufacturers.

A comprehensive approach makes it possible not only to analyze the course of temperature fields, the advancement of the solidification front, and the position of the mushy zone at different points in time, but also to compare the solidification dynamics of the same casting in sand and metal molds.

It has been established that in the metal mold, the cooling of the casting parts occurs more evenly, which is due to the fact that the time of solidification in the metal mold (16 s) is many times shorter than in the sand mold (190 s), so the large temperature difference does not have time to develop. This should contribute to the formation of the denser and fine-grained metal structure (due to rapid cooling) and the minimal development of shrinkage porosity due to the minimal time the metal remains in the state with the temperature below *T_X_*.

For the specific casting geometry and assumptions considered in the present study, the metal mold provides more favorable thermal conditions and a lower analytically estimated tendency toward shrinkage-related problems than the sand mold. This conclusion should be interpreted within the adopted model assumptions and should not be generalized without additional numerical or experimental confirmation for other alloy–mold systems. In this case, the solidification rate can be additionally regulated by such parameters as the mold temperature *T_M_*, the thickness (*δ*_0_) and the coefficient of thermal conductivity (*λ*_0_) of the coating. The model identifies comparative trends and relatively favorable solidification conditions, but it does not provide sufficient grounds for an unconditional claim of complete defect elimination under practical conditions. From the methodological standpoint, the present analytical solution should be regarded as a transparent reference formulation for subsequent numerical verification, especially for assessing axial end effects, temperature-dependent interfacial resistance, and other factors that lie beyond the scope of the current one-dimensional radial treatment.

The authors hope that the presented results of their work will supplement the existing information, expand the understanding of the temperature fields of cast parts, contribute to the development of modeling of solidification processes, and help improve the quality of cast products in general.

### Scope of Applicability and Limitations of the Model

The proposed formulation describes the transient temperature field from the moment of pouring to the complete solidification of the casting under study. It is most appropriate for sufficiently elongated cylindrical castings, in the central region of which radial heat transfer predominates. In the example considered here, the length-to-diameter ratio is 10:1, whereas near the end faces, axial heat losses should be additionally taken into account by numerical methods in order to obtain an accurate description. The present analytical scheme was developed for alloys that solidify over a finite temperature interval and, within the adopted model, do not exhibit a pronounced temperature arrest associated with eutectic transformation. If the eutectic transformation is significant, the description of the final stage of solidification requires modification. In addition, the model predicts temperature histories, solidification-front progression, and the positions of temperature boundaries associated with feeding of the casting; however, these results should be interpreted as indicators of the comparative tendency toward shrinkage defects rather than as direct experimental proof of their formation or complete elimination.

## 6. Conclusions

1. A comprehensive methodology for analytical calculation of temperature fields of castings during the solidification process has been developed, which is divided into two separate modules: solidification in the sand mold and solidification in the metal mold. The methodology is based on the combination of previously available and proven calculation methods (the time of solidification of castings in sand and metal molds τ_T_ and the time of heat removal from overheating τ_over_) and those created by the authors during their own research (the dynamics of temperature changes in the surface *T*_cast_ and the center *T*_center_ of castings, the dynamics of the solidification front advancing from the surface to the center of castings, determination of the distribution of temperature fields in the liquid and solid parts of castings).
2. The temperature field of the cylindrical casting with a diameter of 20 mm made of Al–5%wt.Cu alloy during the solidification process in the sand mold was calculated, as a result of which the following was established: time τ_T_ = 190 s, time of heat removal from overheating τ_over_ = 15 s, surface temperature at the moment of completion of solidification 715 K, maximum temperature difference across the casting cross-section 195 K and it corresponds to the moment of surface cooling exactly to the solidus temperature T_S_, and the total time of existence of the solid–liquid zone of microscopic displacements around the center of the casting of 90 s.
3. The temperature field of the similar casting during the solidification process in the metal (steel) mold was calculated, as a result of which it was established: time τ_T_ = 16 s, time of heat removal from overheating τ_over_ = 3.1 s, surface temperature at the moment of completion of solidification 738 K, maximum temperature difference across the casting cross-section 90 K and it also corresponds to the moment of surface cooling exactly to the solidus temperature *T_S_*, the total time of existence around the center of the casting of the solid–liquid zone of microscopic displacements is 7 s. From this, it is clear that the dynamics of solidification of the casting in the metal mold should ensure the lower analytically estimated tendency toward shrinkage defects under the adopted assumptions. The structure of the methodology allows for its adaptation to other systems, but it is not universal without recalibration of the parameters and boundary conditions.
4. When comparing the dynamics of the temperature difference in the casting, it was found that in the metal mold, this difference is much smaller (90 K), while in the sand mold it is 195 K, which also contributes to improving the conditions for solidification, structure formation and formation of metal properties.
5. The developed analytical methodology is not limited to the specific Al–5 wt.% Cu casting examined here; however, its transfer to other alloy–mold systems requires recalibration of thermophysical, interfacial, and geometric parameters and, where necessary, numerical refinement. Its main contribution lies in the mathematical procedure for reconstructing the time evolution of boundary temperatures, solidification-front progression, and transient temperature gradients under clearly stated assumptions.

## Figures and Tables

**Table 1:** The initial data used for the calculations.

Calculation Parameter	Marking	Units of Measurement	Numerical Value
Alloy			
Pouring temperature	*T_pour_*	K	1123
Liquidus temperature	*T_L_*	K	910
Solidus temperature	*T_S_*	K	780
Specific heat of solidification of the alloy	*Q*	kJ/kg	360
Effective heat of solidification of the alloy (which takes into account heat release due to cooling from temperature T_L_ to temperature T_S_)	*Q_ef_*	kJ/kg	535
Density of the alloy in the liquid state	*ρ_L_*	kg/m^3^	2700
Specific heat capacity of the alloy in the liquid state	*C_L_*	J/(kg·K)	1200
Specific heat capacity of the alloy in the solid state	*C_S_*	J/(kg·K)	1000
Thermal conductivity coefficient of the alloy in the liquid state	*λ_L_*	W/(m·K)	102
Thermal conductivity coefficient of the alloy in the solid state	*λ_S_*	W/(m·K)	215
Thermal diffusivity coefficient of the alloy in the liquid state	*a_L_*	m^2^/s	3.15·10^−5^
Thermal diffusivity coefficient of the alloy in the solid state	*a_S_*	m^2^/s	6.7·10^−5^
Thermal accumulation coefficient of the alloy	*b_S_*	W·s^1/2^/(m^2^·K)	26,000
*Sand casting mold*			
Initial mold temperature	*T_M_*	K	293
Heat accumulation coefficient	*b_M_*	W·s^1/2^/(m^2^·K)	950
Specific heat capacity of the mold	*C_M_*	J/(kg·K)	1100
Density	*ρ_M_*	kg/m^3^	1600
*Metal (steel) casting mold*			
Initial mold temperature	*T_M_*	K	523
Heat accumulation coefficient	*b_M_*	W·s^1/2^/(m^2^·K)	17,540
Specific heat capacity of the mold	*C_M_*	J/(kg·K)	750
Density	*ρ_M_*	kg/m^3^	7500
Mold wall thickness	*l_M_*	m	0.03
Thermal conductivity coefficient of the coating on the mold surface	*λ*_0_	W/(m·K)	0.350
Thickness of the coating on the mold surface	*δ*_0_	m	0.0002

## Data Availability

The original contributions presented in this study are included in the article. Further inquiries can be directed to the corresponding author.
